# How do Clinicians Use Electronic Health Records for Respiratory Support Decisions? A Qualitative Study in Critical Care

**DOI:** 10.1055/a-2521-2557

**Published:** 2025-02-17

**Authors:** Tianyi Zhang, Jarrod Mosier, Vignesh Subbian

**Affiliations:** 1 College of Engineering, University of Arizona, Tucson, Arizona, United States; 2 College of Medicine- Tucson, University of Arizona, Tucson, Arizona, United States

**Keywords:** electronic health record, critical care, respiratory support, clinical decision support, qualitative study

## Abstract

**Objectives** Selecting appropriate respiratory support in critical care is complex, and some decisions require information that may be unknown when the treatment assignment is necessary. Digital technologies such as electronic health records (EHR) are essential components in critical care medicine to support respiratory support care delivery and management of patients with respiratory failure. However, there are limited studies on EHR use that enable clinical decisions related to respiratory support. The objective of this study is to understand how clinicians use EHRs for their decision-making related to respiratory support in intensive care units (ICUs).

**Methods** Using a socio-technical systems approach, we conducted nine observations with nine different care teams for 35 hours at two ICUs within a large academic hospital system. We created a journey map to illustrate clinicians' respiratory support decision-making processes. We identified barriers related to decision-making processes within the ICU socio-technical work context and characterized them based on macro-cognitive functions to derive themes that can capture the decision-making patterns associated with EHR use.

**Results** Our analysis identified three overarching themes that represent clinicians' use of EHR for their respiratory support decisions: (1) fragmented information and tasks for individual sensemaking; (2) EHR workarounds for collaborative decision-making; and (3) interruptive order entry and order execution. These three themes represent three major sequential stages (i.e., before, during, and after morning rounds) related to clinicians' respiratory support decision-making processes, and their interaction with EHR significantly varies between stages.

**Conclusion** Our findings reflected different EHR use patterns before, during, and after morning rounds for decision-making related to respiratory support. These findings indicated potential opportunities for diagnostic clinical decision support (CDS) to facilitate respiratory support decisions.

## Background and Significance

Respiratory support decisions in critical care are challenging and time-sensitive for patients with respiratory failure, as clinicians need to promptly select the modality of respiratory support (e.g., selecting invasive mechanical ventilation vs. noninvasive respiratory support [NIRS] via a mask or specially designed nasal cannula). Additionally, they must determine optimal settings for that support and conduct follow-up assessments to identify the need for adjustments to the strategy or support. 1 This decision-making process requires clinicians to review and interpret large amounts of electronic health record (EHR) data, while also accounting for relevant device attributes such as oxygen flow rate, ventilator, noninvasive ventilator, or high flow delivery system settings, and appropriate length of time on a certain respiratory support modality. Deciding on such a patient-specific respiratory support plan in a timely fashion is critical based on evidence that NIRS is most effective when started early 2 3 and delayed intubation following the failure of NIRS could lead to an increased risk of morbidity and mortality. 4 5 6 7 8

Since the Health Information Technology for Economic Clinical Health Act in 2009, the Centers for Medicare and Medicaid Services have incentivized healthcare organizations to adopt and meaningfully use EHR technology. 9 EHRs are intended to leverage key functionalities such as clinical decision support (CDS), computerized order entry, and health information exchange to improve quality, safety, and efficiency and reduce the cost of healthcare. 10 11 In the realm of critical care medicine, EHRs are central to the coordination and delivery of care among intensive care unit (ICU) teams, as they accrue large volumes of clinical data needed for intensivists' decision-making. 12 13 14

Some studies have attempted to integrate different types of CDS (e.g., alert support, protocol/procedure support, management support) into their EHR systems to facilitate respiratory support decisions in adult critical care. These efforts have demonstrated the potential to yield positive impacts on respiratory support management. For example, monitoring mechanically ventilated patients and alerting bedside providers via paging notifications can decrease the chances of ventilator-induced lung injury. 15 Another study showed that the use of CDS in their EHR improved adherence to the low tidal volume protocol. 16 In terms of clinical management support, studies have found that CDS can appropriately select inspired oxygen fraction based on automatic frequent assessments of patients 17 18 and predict weaning readiness earlier than intensivists. 19 However, there are no studies related to computerized CDS for providing diagnostic support that facilitates the decision-making of selecting appropriate respiratory support for patients with acute respiratory failure, particularly in the ICU context.

## Objectives

In this study, we sought to understand how intensivists use EHR and other means, including any CDS tools embedded within EHR, for their decision-making related to respiratory support. We previously developed a rule-based phenotyping algorithm that can reliably identify and classify patients with acute respiratory failure, 20 21 as well as a model that can predict patients at risk for failing a non-invasive strategy. 8 We ultimately intend to build and implement CDS tools based on our phenotyping algorithm and predictive model that can provide diagnostic support and aid intensivists' decisions in selecting an appropriate respiratory support strategy. However, before doing so, we recognize potential challenges associated with the design and implementation of CDS, including issues such as information overload, information fragmentation, and interruption to workflow and communication. 22 23 To proactively address these unintended consequences, we conducted an observational investigation into clinical workflows, including interactions with EHR, within the ICU, with a focus on decision-making processes related to respiratory support strategies.

## Methods

This study was informed by the socio-technical systems approach, specifically the systems engineering initiative for patient safety (SEIPS) framework. 24 25 26 The SEIPS model has supported the understanding and evaluation of complex sociotechnical systems and has been used to frame the research design and analysis. The SEIPS model depicts a sociotechnical work system with five interacting components (i.e., the person(s), tasks, tools and technologies, organization, and environment) that produce work processes, which affects the system outcome. In our study, the SEIPS model helped us capture each work system component during observational data collection, which yielded rich observational data and ensured a holistic examination of the work system (i.e., the ICU) where clinicians interact with the EHR and CDSs to finalize their respiratory support related decision-making. This study was approved by the University of Arizona Institutional Review Board (Protocol #: *2011215104A001* ).

### Research Setting

This observational study was conducted in two ICUs within a single university-affiliated hospital system located in the Southwest United States. One ICU is located in a central part of the city within a regional Level I Trauma Center with 649 licensed hospital beds including 96 intensive care adult beds. Another comparatively smaller ICU is located on the city outskirts with 245 licensed beds including 12 intensive care adult beds. Each ICU has distinct nursing and resident staffing, while the faculty and fellows for both ICUs are from the same department (Department of Medicine). The entire health system uses a commercial, enterprise EHR system (Oracle Cerner, Oracle Corporation).

### Participants

The study participants included attending physicians responsible for overseeing all care decisions, as well as fellows and residents who actively participated in clinical decision-making as members of ICU care teams. Although ICU care teams often also included pharmacists, medical students, and sometimes nursing students, they were not the focus of our observations because they were not involved in the clinical decision-making processes for respiratory support. Our study participants were recruited through convenience sampling with no specific exclusion criteria, with critical care medical directors providing access to the ICU staffing schedule and facilitating outreach to care teams. This sampling method ensured the inclusion of various combinations of attendings and fellows while respecting the operational constraints of the ICU. Participating attending physicians and fellows were contacted in advance to obtain permission to shadow and observe their care teams. All informed consent forms were distributed and signed via REDCap.

### Procedure

We conducted a 6-hour pilot observation to become familiar with the ICU environment and workflow. We identified that clinicians typically coordinate their respiratory support plan for admitted patients during morning rounds outside patient rooms starting at around 8 am each day. However, fellows and residents start their shifts between 5:30 and 6:00 am to prepare for the morning rounds. Preparation involves a series of tasks that are supported by the EHR. Attending physicians often arrive around 7:00 am to prepare for morning rounds. Therefore, we began the formal observations when fellows and residents started their preparation at their workstations around 5:30–6:00 am and ended the observations when the care team concluded their morning rounds and completed residual tasks from morning rounds, such as order entry.

At the beginning of each formal observation, the observer (TZ) provided a brief introduction of the study to the care team, briefly conversed with the clinical fellow to understand the general situation of the day, and noted patient cases that would potentially involve respiratory support decisions. During observations, the observer followed care teams, took notes, and conducted opportunistic interviews to clarify what was observed, when possible, without interfering with the normal clinical workflow. After each observation, handwritten field notes were transcribed into digital notes, which were then organized, expanded, and annotated with additional contextual information and comments on the same day. We continued collecting observational data until no new themes emerged and there was redundancy in the behaviors and patterns observed across the study sites.

### Analysis

We first created a journey map to organize clinicians' respiratory support decision-making processes and depict how clinicians interact with other work system factors over time. Journey maps, unlike other types of process maps, illustrate processes as well as the relevant change of work system factors and outcomes over time, which are frequently employed to operationalize the SEIPS concept and to pinpoint issues or trends that require attention in a process or system. 27 After creating the journey map, we conducted a thematic analysis to identify and analyze repeated patterns and construct themes. 28 Specifically, we identified barriers within the ICU socio-technical work context that are related to EHR use during the respiratory support decision-making processes. We further aggregated and categorized these barriers in terms of their macrocognitive functions and systematically compared them to derive themes that can capture the patterns and trends. One investigator (TZ) initially reviewed and coded each transcript, and another investigator (VS) reviewed the codes and coding structure. Through multiple iterations, all codes and themes were thoroughly discussed and refined until disagreements were solved and the final consensus was reached with the approval of the subject matter expert (JM). We used the following methods to ensure research quality and rigor regarding credibility, transferability, dependability, and confirmability. 29 30 Specifically, the strategies included peer debriefing and member checks for credibility; purposeful sampling for transferability; peer examination and triangulation for dependability; self-reflections throughout the process, and multiple rounds of coding for confirmability.

## Results

We conducted nine formal observations between August 2022 and October 2022 for a total of 35 hours. Of these, six observations were at the central medical ICU (site A) and three observations were at the other ICU (site B; see Table 1 ). The observed care teams were led by nine different attending physicians across the two ICU sites.

**Table 1:** Characteristics of participants and observations sites

Observation	ICU site	Patient cases	Care team a		
			Size	Male	Female
1	B	6	6	4	2
2	A	10	8	5	3
3	B	5	7	6	1
4	A	4	10	7	3
5	A	8	9	6	3
6	A	6	10	6	4
7	A	6	10	7	3
8	B	4	9	7	2
9	A	13	8	6	2
*n* = 9	A = 6, B = 3	*n* = 62	*n* = 9	54 (70.1%)	23 (29.9%)

a The care teams consist of one attending physician, one fellow, and several residents.

**Fig. 1:**
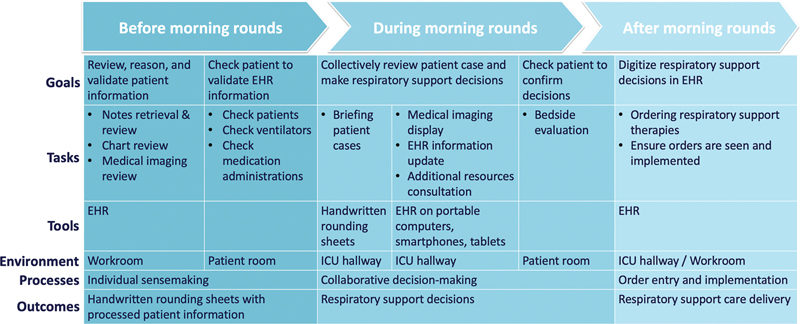
Journey map of clinicians' respiratory support decision-making processes.

The journey map (see Fig. 1 ) depicted three major sequential stages related to clinicians' respiratory support decision-making processes (i.e., before morning rounds, during morning rounds, and after morning rounds). We identified three overarching themes (see Table 2 ) that represent clinicians' use of EHR for their respiratory support management decisions: (1) fragmented information and tasks for individual sensemaking before morning rounds; (2) EHR workarounds for collaborative decision-making during morning rounds; and (3) interruptive order entry and order execution after morning rounds.

**Table 2:** Themes and descriptions of EHR use for respiratory support decisions at different stages

Stages	Themes	Description
Before morning rounds	Fragmented information and tasks for individual sensemaking	Information seeking process for sensemaking consisted of fragmented tasks and information • Difficulty in retrieving relevant clinical notes • High variance of EHR use when retrieving relevant structured, quantitative data • Forced screen transitions • Interruptive CDS encounters • Additional communication to validate EHR information
During morning rounds	EHR workarounds for collaborative decision-making	EHR workarounds included handwritten rounding sheets, smartphones, portable computers, and tablets. The following are four occasions where portable computers were used as a workaround • To provide missing patient data or correct incorrect data during resident's briefing • To resolve conflicting information between EHR and the real-world • To access and display imaging results (e.g., chest X-rays) • To complete simple order entries
After morning-rounds	Interruptive order entry and order execution	• Clinicians tended to delay entering complicated orders as the order entry was interruptive to their care delivery • Order entries were sometimes disrupted by system alerts • Extra team effort was needed to mediate order entries and executions due to lack of EHR system display consistency

Abbreviations: CDS, clinical decision support; EHR, electronic health records.

### Theme 1: Fragmented Information and Tasks for Individual Sensemaking Before Morning Rounds

Before morning rounds, clinicians started their shift by aggregating and making sense of patient data and information in the EHR that will be needed for briefing at morning rounds. This sensemaking process is highly dependent on the individual interaction with the EHR at their workstations. There are several common patterns observed among individual interaction with the EHR: (1) difficulty in retrieving relevant clinical notes; (2) high variance in EHR use for retrieving relevant structured, quantitative data; (3) forced screen transitions; (4) interruptive alerts and other CDS encounters; and (5) additional communication to validate EHR information. These common patterns embody fragmented tasks related to EHR use that are required for individual sensemaking.

First, it was observed that most clinicians started the sensemaking process by checking notes such as physician progress notes and consultation reports in the documentation section. The major complaint observed during this stage was the difficulty in retrieving needed patient information from notes because pertinent patient information was often buried in repetitive notes in various documents.

Second, there was a high variance in EHR use for retrieving structured data. For example, to find the ventilator settings, some clinicians checked the blood gas settings under the results interface, some clinicians found it in the interactive view interface, and some clinicians found the ventilator setting in the assessment section. However, the ventilator setting data were not consistently displayed in those three interfaces as one contained more up-to-date ventilator setting data than another. Two fellows postulated that such variance in EHR use in finding the ventilator settings could be attributed to a lack of awareness of the nuances in the ventilator data display amongst residents.

Third, constant screen transitions were prevalent and did not conform to clinicians' sensemaking habits. To understand physician progress notes (e.g., why was this patient not intubated?), clinicians had to “pull” related data from multiple sections in the EHR, yet the system neither allowed the simultaneous display of two or more sections nor provided a semantically integrated view of various data artifacts. To seek external decision support (e.g., logging in to UpToDate), clinicians had to transition the screen out of the EHR interface. Additionally, it was noted that the EHR system only allowed two open patients' charts on the tab from the patient list, which caused additional navigation when attending phyisicians wanted to look at more than two patients. Forced screen transitions were related to fragmented patient data and information across many domains (e.g., results, documentation, interactive view, and medication records), which prompted various workarounds among clinicians. For instance, some clinicians used their smartphones or tablets as the second screen, whereas some clinicians printed paper copies of notes and compared them against digital/EHR data. These workarounds also extended into supporting later collaborative decision-making elaborated in Theme 2.

Last, additional communication was required to validate EHR data and information as it may be outdated, inconsistent, and incomplete. For example, as clinicians knew that EHR may not capture up-to-date overnight data, they talked with bedside nurses to make sure data on the ventilator matched the EHR data. This was due to a lack of effective interoperability between medical equipment such as ventilators and EHR for automated data collection. Another example is that a clinician detected an anomaly in EHR data and found the patient was actually on mechanical ventilation, but the vent order was not documented. The required walking and talking often happened in the middle of their EHR interaction and were interruptive to their EHR use.

### Theme 2: EHR Workarounds for Collaborative Decision-Making During Morning Rounds

While clinicians' sensemaking was supported by their EHR interaction before morning rounds, the collaborative decision-making during morning rounds outside patient rooms depends on limited EHR use via portable computers and other peripheral devices such as smartphones, tablets, or paper records such as handwritten rounding sheets. The observed morning rounds usually involved attending physicians with their patient lists listening to residents briefing on patients based on their rounding sheets. Then, attending physicians led the discussion of the assessment plan with the team, during which attending physicians requested to see chest imaging on a portable computer and residents were instructed to consult external resources (e.g., search for the five World Health Organization pulmonary hypertension groups on smartphones) or calculate indices (e.g. calculate the ratio of SpO _2_ /FiO _2_ to respiratory rate score using an online calculator) needed for respiratory support decision-making. Before finishing the assessment and plan discussion, some of the care teams entered the patient room to perform bedside evaluations. There was no interaction with EHR observed in the patient room.

The majority of the care teams, except a few attending physicians, depend on handwritten rounding sheets that were derived from the sensemaking of EHR data and information before morning rounds during their collaborative decision-making outside patient rooms. Handwritten rounding sheets as a workaround for EHR use were structured differently among individual physicians; however, what they contained were similar—a semantically integrated display of subjective data (e.g., overnight events), objective data (e.g., vitals, labs, physical exam), and assessment and plan.

Two clinicians (one attending and one fellow) were observed using tablets (e.g., Microsoft Surface, and Apple iPad) during morning rounds. However, there were several drawbacks associated with their EHR use on tablets. For example, they used the web-based EHR portal on tablets, which was not completely compatible with tablet platforms (e.g., they could not zoom in or zoom out when looking at imaging on tablets). In addition, they had no place to temporarily put away their tablets while interacting with patients because the current ICU work environment does not support the use of tablets.

Although most members of the care teams depended on handwritten rounding sheets, two care team members were typically designated as portable computer users for any interaction with EHR. There were four occasions that portable computers were needed for further EHR interactions to continue supporting collaborative decision-making: (1) providing missing patient data or correcting incorrect data during resident's briefing; (2) resolving conflicting information between EHR and the real world; (3) accessing and displaying chest imaging; (4) completing simple order entries.

First, residents sometimes missed important clinical data or reported incorrect data in their morning rounds briefing. In this case, residents with portable computers supplemented missing data and corrected incorrectly reported data. For example, an attending felt some data were “off” in the resident's briefing and requested validation from residents using portable computers, who later confirmed that the patient's blood pressure did not increase.

Second, as EHR data can be not up-to-date, incomplete, or incorrect, 31 residents with portable computers needed to update the system outside the patient room. For example, the attending physician detected an inconsistency in the medication summary, went into the patient's room, and found that the patient was not on the said medication.

Third, portable computers are the only channel for imaging display to the care team during morning rounds. Since chest imaging is an important component of respiratory support decision-making, attending physicians always requested displays of the chest imaging during the care team discussion. Yet, these images can only be satisfactorily viewed on a computer and other workarounds such as tablets were suboptimal for display.

Last, residents or pharmacists with portable computers sometimes complete simple orders outside patient rooms. It allows for quick order entry that minimizes delays and interruptions to their later workflow.

### Theme 3: Interruptive Order Entry and Order Execution After Morning Rounds

Respiratory support decisions finalized through collaborative decision-making outside patient rooms were electronically documented and ensuing actions were entered as orders. The digitization of decisions into the EHR was intended for multiple purposes, such as billing, facilitating collaborative teamwork, and streamlining clinical workflow 32 ; however, the process of digitizing decisions was disruptive to teamwork and clinical workflow. While residents with portable computers could quickly enter simple orders outside patient rooms, for certain complicated orders, clinicians tended to delay those order entries after they finished morning rounds. Nevertheless, clinicians' care delivery did not stop after morning rounds and the care teams faced a dilemma—should we go see another patient or should we go back to the workroom to enter orders? Hence, order entries were seen as interruptions to their care delivery processes. In addition, entering orders into the EHR could prompt system alerts that disrupt the order entry. For example, a bi-level positive airway pressure (BiPAP) protocol CDS was built into the EHR during the COVID-19 pandemic, as such placing an order for BiPAP requires the completion of a BiPAP advisor checklist first. While the intended purpose of the BiPAP protocol CDS was to ensure patient safety, the way it functioned as a post hoc decision check was interruptive to clinicians' workflow and decision-making processes. Last, order execution sometimes required extra teamwork effort to mediate because EHR interfaces were not consistently and uniformly displayed to different care providers. For example, the orders entered by clinicians sometimes were not seen by nurses or respiratory therapists on their EHR interfaces. The clinician had to follow up in person to ensure that orders in the EHR were seen and implemented.

## Discussion

Based on our observations, we identified three major stages of respiratory support-related decision-making: individual sensemaking before morning rounds, collaborative decision-making during morning rounds, and interruptive order entry after morning rounds. The extended observation period in this study, spanning from pre-morning rounds to after-morning rounds, was critical for capturing the entire decision-making journey from early stages to finalization of decisions.

Our analysis showed patterns and dynamics associated with EHR use (e.g., the extent, timing, and purpose of EHR use) significantly vary at these stages. Some of our findings regarding EHR use during morning rounds are consistent with the previous study. 33 For example, morning rounds depend on portable computers, smartphones, tablets, and handwritten rounding sheets, which are workarounds for accessing EHR information. However, our extended observation period also uncovered EHR use patterns before and after morning rounds that are rather different from those during morning rounds. Contrary to relying on EHR workarounds during morning rounds, clinicians spent more time directly interacting with the EHR before and after morning rounds, during which suboptimal EHR interaction experience was observed. Specifically, before morning rounds, clinicians' sensemaking processes were frequently disrupted by challenges in retrieving and verifying EHR data as well as the absence of adequate care context documented in the EHR. A prior study pointed out that while most EHRs document treatment plans, they do not necessitate explicit documentation of the patient's care context, which could explain the underlying reasons behind clinicians' treatment decisions. 34 We observed that, due to the lack of care context documentation in the EHR, residents sometimes could not understand why the established patients were assigned a respiratory support treatment or fellows and attendings sometimes could not figure out why newly admitted patients were on certain respiratory support treatment based on the existing information in the EHR. To prepare and facilitate collaborative respiratory support decision-making at morning rounds, clinicians have to seek information within the current EHR display and semantically integrate patient care context on their handwritten rounding sheets. However, once respiratory support decisions are finalized, clinicians must document their notes and orders back into the EHR, during which the rationale behind their decisions may not be fully captured. In light of the issues with the current EHR display, a recent effort has been devoted to designing a semantically integrated display for chart review. 35 Nevertheless, our findings highlight the need to redesign documentation and order entry to integrate patient care context.

At present, there is a notable gap in the availability of diagnostic CDSs specifically tailored to respiratory support-related decision-making. Although a few studies 15 16 17 18 19 have attempted to integrate different types of CDSs (e.g., alert support, protocol/procedure support, management support) into their EHR systems and reported potential positive effects on respiratory support in adult critical care, we did not observe any similar CDSs being locally implemented and used. In addition, no diagnostic CDS related to respiratory support decision-making was observed. This is not uncommon because diagnostic CDSs are less widely accepted and implemented than other forms of CDSs. 36

To inform the design and implementation of diagnostic CDSs that facilitate respiratory support decisions and enable utilization, our findings can be further interpreted using the five Rights of CDS framework. 37 Specifically, our study identified the “right time within the clinicians' workflow” to incorporate respiratory support-related diagnostic CDS. For a diagnostic CDS to better aid respiratory support decisions in ICUs, it would be more meaningful if they appeared early in the decision-making process such as the individual sensemaking stage before morning rounds for several reasons. First, the individual sensemaking stage is essential to the subsequent collaborative decision-making yet it can be challenging for residents due to insufficient experience. CDSs can aid clinicians' information-seeking and sensemaking in multiple ways, such as facilitating notes or data retrieval and automating the calculation of some important indices. Second, collaborative decision-making is mostly based on handwritten notes or tablets with the occasional support of portable computers for the display of X-ray imaging and smartphones for references and calculation. This process is primarily conducted through in-person communication, during which direct interaction with EHR could be interruptive even for necessary uses (e.g., request for chest X-ray display). Third, the appearance of CDS during order entry would not likely aid decision-making because decisions were already made collectively, and it can instead be interruptive to the order entry process.

## Limitations

This study has several limitations. First, although we conducted observations at two ICUs, these ICUs are two locations within the same hospital that both implement the same EHR system. Hence, not all of our findings may be generalizable to other hospital settings with other EHR systems. Second, we note that the sizes of the two observed ICUs are different, but our analysis did not identify systematic variation in clinicians' EHR use that could be attributed to unit size. This may be due to several factors: (1) both units are part of the same health system, with shared protocols, workflows, and culture; (2) attendings often rotate between the two sites, ensuring consistency in decision-making approaches, and (3) the observed care team sizes and composition during the study period are consistent with normal practices in both units, which likely minimized the potential influence related to the unit size. However, differences in unit sizes may have a more pronounced impact on other healthcare systems. Third, our observations were conducted by one observer. This, however, may provide more consistency and standardization in our 35 hours of observational data collection. 38 Fourth, our sample size is relatively small as we only observed nine care teams. However, the number of observations was not finalized until we had reached data saturation. Lastly, the presence of the observer might affect how clinicians present themselves in the study, which is a common concern in qualitative studies. To mitigate the potential impact, we communicated clearly with clinicians before every observation that the observation was not assessing their performance but rather observing their interaction with digital tools such as the EHR during their clinical decision-making process.

## Conclusion

We identified three themes that represent current EHR use patterns in the decision-making process: fragmented information and tasks for individual sensemaking before morning rounds; EHR workarounds for collaborative decision-making during morning rounds; and interruptive order entry and order execution after morning rounds. The uncovered dynamic patterns of EHR use present opportunities for designing and developing a useful diagnostic CDS that supports decision-making related to respiratory support. Additionally, it informs the implementation of such CDS on how to be optimally integrated into EHR workflows and decision-making processes.

## Clinical Relevance Statement

Respiratory support decisions in critical care are challenging and time-sensitive. We previously developed a rule-based phenotyping algorithm that can reliably identify and classify patients with acute respiratory failure based on respiratory support strategy, as well as a model that can predict patients at risk for failing a non-invasive strategy. This qualitative study provides insights into the design, implementation, and integration of such diagnostic CDS in EHR that would facilitate clinicians' decisions in selecting an appropriate respiratory support strategy.

## Multiple Choice Questions

1. Which of the following is not a common pattern observed in the EHR use before morning rounds in the ICU?
  - Difficulty in retrieving relevant clinical notes
  - Difficulty in finding relevant structured patient data
  - Forced screen transitions
  - Interruptive alerts or CDS encounters
  **Correct answer** : b. Our study shows that clinicians can find the relevant structured patient data through different interfaces; however, data was not identically displayed among these interfaces as some display more up-to-date data than others, and some residents may not be aware of the nuances.
2. Why portable computers were needed for further EHR interactions to continue supporting collaborative decision-making during the morning rounds?
  - To provide missing patient data or update data during resident's briefing
  - To access and display chest imaging
  - To quickly complete simple orders
  - All above
  **Correct answer** : d. Although the majority of the care team relied on their handwritten notes to support collaborative decision-making during morning rounds, a few senior residents had to use portable computers to support data input (e.g., order entry or data update) and satisfactorily display X-ray imaging, which is not supported by other EHR workarounds.
